# Tumor angiogenesis and anti-angiogenic therapy in malignant gliomas revisited

**DOI:** 10.1007/s00401-012-1066-5

**Published:** 2012-11-11

**Authors:** Karl H. Plate, Alexander Scholz, Daniel J. Dumont

**Affiliations:** 1 Institute of Neurology (Edinger Institute), Frankfurt University Medical School, Frankfurt, Germany; 2 Sunnybrook Research Institute, Sunnybrook Health Science Center, Toronto, ON Canada

## Abstract

The cellular and molecular mechanisms of tumor angiogenesis and its prospects for anti-angiogenic cancer therapy are major issues in almost all current concepts of both cancer biology and targeted cancer therapy. Currently, (1) sprouting angiogenesis, (2) vascular co-option, (3) vascular intussusception, (4) vasculogenic mimicry, (5) bone marrow-derived vasculogenesis, (6) cancer stem-like cell-derived vasculogenesis and (7) myeloid cell-driven angiogenesis are all considered to contribute to tumor angiogenesis. Many of these processes have been described in developmental angiogenesis; however, the relative contribution and relevance of these in human brain cancer remain unclear. Preclinical tumor models support a role for sprouting angiogenesis, vascular co-option and myeloid cell-derived angiogenesis in glioma vascularization, whereas a role for the other four mechanisms remains controversial and rather enigmatic. The anti-angiogenesis drug Avastin (Bevacizumab), which targets VEGF, has become one of the most popular cancer drugs in the world. Anti-angiogenic therapy may lead to vascular normalization and as such facilitate conventional cytotoxic chemotherapy. However, preclinical and clinical studies suggest that anti-VEGF therapy using bevacizumab may also lead to a pro-migratory phenotype in therapy resistant glioblastomas and thus actively promote tumor invasion and recurrent tumor growth. This review focusses on (1) mechanisms of tumor angiogenesis in human malignant glioma that are of particular relevance for targeted therapy and (2) controversial issues in tumor angiogenesis such as cancer stem-like cell-derived vasculogenesis and bone-marrow-derived vasculogenesis.

## Introduction

In his landmark 1971 publication, Folkman [41] developed the idea that tumor growth is angiogenesis dependent and described for the first time the potential prospects of anti-angiogenic cancer therapy. From thereon, it took 13 years before fibroblast growth factor (FGF)-2, the first heparin-binding angiogenic growth factor, was identified and 18 years before vascular endothelial growth factor (VEGF), which by now has turned out to be the single most important angiogenesis factor in both health and disease, was described (for review see [38]). Napoleone Ferrara, one of the discoverers of VEGF, developed monoclonal antibodies to VEGF [70] that blocked tumor growth in vivo [71] and that paved the way for the development of bevacizumab, a monoclonal anti-VEGF antibody that is now used in a large number of clinical cancer trials, including glioblastoma, where anti-angiogenesis represents one treatment arm [104, 131]. It is now evident that the entire process of tumor-induced angiogenesis appears to be far more complex than initially envisioned [15]. Moreover, the idea that blockade of tumor angiogenesis is able to inhibit tumor growth in vivo has been confirmed in principal in both experimental and clinical settings; however, current evidence suggests that cancer cells are able to circumvent anti-angiogenic therapy and develop resistance to targeted mono-therapy [7, 129].

## Cell biology of tumor angiogenesis

**Fig. 1:**
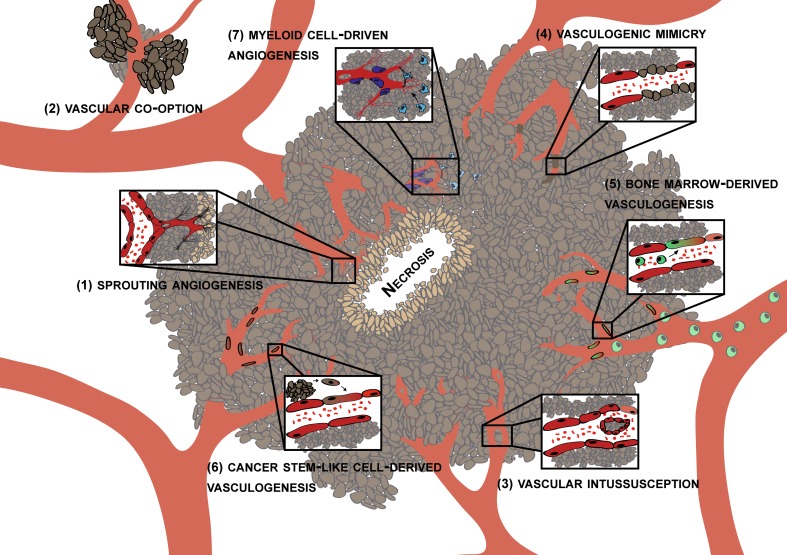
Potential mechanisms of glioma angiogenesis. Currently, (1) sprouting angiogenesis, (2) vascular co-option, (3) vascular intussusecption, (4) vasculogenic mimicry, (5) bone marrow-derived vasculogenesis, (6) cancer stem-like cell derived vasculogenesis and (7) myeloid cell-driven angiogenesis are all considered to contribute to tumor angiogenesis. However, some of these mechanisms have initially been described in developmental angiogenesis and their relative contribution and relevance in human brain cancer is unclear (see text)

In a recent *Cell* snapshot Jain and Carmeliet listed six different principal cellular mechanisms under the heading of tumor angiogenesis. These include (1) classical sprouting angiogenesis, (2) vascular co-option, (3) vessel intussusception, (4) vasculogenic mimicry, (5) bone marrow derived vasculogenesis and (6) cancer stem-like cell derived vasculogenesis [62]. Another important mechanism of tumor angiogenesis is angiogenesis driven by blood derived infiltrating myeloid cells, a process that recently received considerable attention. Whether the above listed mechanisms of tumor angiogenesis (for overview see Fig. 1) are all operational in human glioblastoma is not entirely clear. Here, we briefly discuss the above mentioned angiogenesis mechanisms and review the existing evidence for their role in glioma biology.

**Fig. 2:**
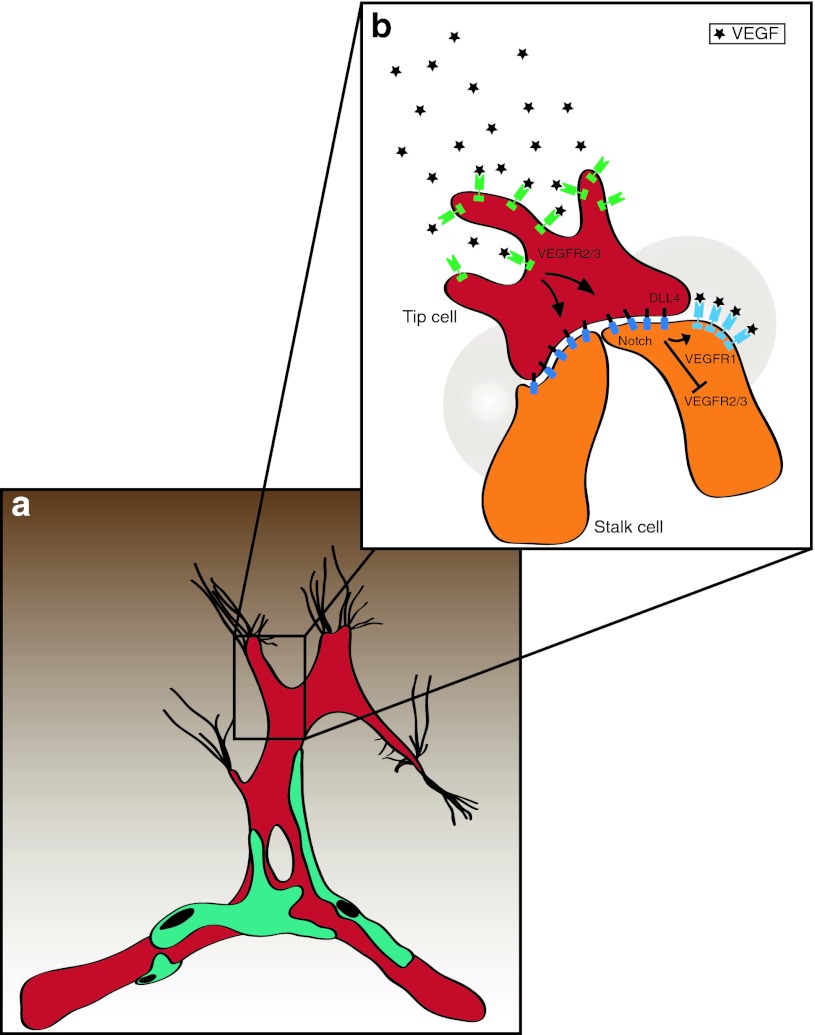
VEGF and Notch regulate tip and stalk cell dynamics during vascular sprouting. **a** Angiogenic sprouting is initialized by VEGF signaling and spatially and temporally regulated by a VEGF gradient (*brown*). Endothelial tip cells exhibit numerous filopodia extensions sensing the environment for migration cues and are devoid of pericyte coverage (*turquois*). **b** Blowup showing the induction and maintenance of tip versus stalk cell identity. Tip cells are induced by VEGF (*black*) that binds to VEGFR2/3 (*green*) localized on the filopodia. VEGF-signaling in tip cells up-regulates Dll4 (*black*) and Notch1 (*blue*) ligand. Dll4–Notch signaling will in turn modulate VEGFR2/3 in adjacent cells, thereby inducing the stalk cell phenotype. Furthermore, VEGFR1 (*light blue*) becomes up-regulated and acts as a VEGF decoy receptor (*shaded circular area*) and helps maintaining the VEGF gradient

*Angiogenesis*, the sprouting of capillaries from pre-existing vessels, has since long been considered the principal mechanism of brain vascularization, both during development and in brain cancer [80, 81, 111, 121]. A vascular sprout that is led by a filopodia-rich tip cell migrates toward an angiogenic stimulus (often VEGF) that is produced by tumor cells. Following the tip are a group of cells, entitled stalk cells, that divide, which promotes the elongation of the sprout (Fig. 2). Tip cells from two distinct sprouts may fuse to yield a larger sprout, a process that is thought to be modulated by the action of macrophages on these activated endothelial cells (Fig. 4). Thereafter, the newly formed vessel remodels to form a vascular lumen (that permits blood flow) and to attract mural cells that stabilize the vessel (reviewed in [16, 143]). Current evidence suggests that vascular sprouting represents a major mechanism of tumor angiogenesis. Analysis of cell division in glioblastoma, by MIB-1 staining, suggests that there are a considerable number of vascular endothelial cells undergo cell division suggesting that these cells may represent stalk cells. In addition to this, molecules typically expressed on endothelial tip cells such as VEGFR-2, Neuropilin-1, Angiopoietin-2 (Ang-2), Integrin-ß1 and others have been described to be present in glioma vessels in situ [11, 31, 114], implying that tip and stalk cell phenotypes co-exist in the glioblastoma vasculature.

*Vascular co*-*option* describes the infiltration of tumor cells into normal tissue and the adoption of the pre-existing vasculature [56]. When one takes this into consideration, vascular co-option may be viewed as part of the invasive phenotype that is intrinsic to all diffuse gliomas—rather than an active vascular process. Invoking the process of vascular co-option aligns well with the known migratory pattern of tumor cells along vessels which has been observed in numerous experimental glioma models in rodents. Vascular co-option may facilitate the infiltration of human gliomas. Of note, anti-VEGF treatment may drive glioma cells to utilize the co-option pathway thereby circumventing the impact of anti-VEGF treatment resulting in an increase in the number of migrating glioma cells, that may use pre-existing vessels as scaffolds for their migration pathways (see below).

*Vessel intussusception* describes the formation of a new vessel by vascular invagination, intra-luminar pillar formation and splitting. Vascular intussusception has initially been described in physiological vascular development [32] but more recently has been expanded to experimental tumors. It has been suggested that sprouting angiogenesis may switch to vascular intussusception to allow rapid development of new vessels [119]. The molecular mechanisms that drive vascular intussusception are currently poorly understood and whether intussusception occurs or plays a role in human glioma or tumor biology in general is currently unclear.

*Vasculogenic mimicry* is defined as a process where tumor cells replace endothelial cells and form a vessel with a lumen This phenomenon was first described (mainly based on PAS staining) in a subset of aggressive uveal melanomas [95] and later in other types of cancer but its overall occurrence and significance, if any, is highly controversial. Moreover, it is unclear whether vasculogenic mimicry represents an active process (e.g., cancer cells actively forming the vascular lumen) or whether vasculogenic mimicry is a consequence of vessel regression. Vessel regression is part of the vascular remodeling process that takes place in development but can also occur in tumors (Fig. 2a). During development, a normal and well-orchestrated realignment of the balance between growth/survival signals such as high levels of Ang-2 together with low levels of VEGF leads to vessel regression and endothelial cell apoptosis causing vessel pruning and vessel remodeling [50]. A similar situation (high levels of Ang-2 combined with low levels of VEGF) can occur in cancer after bevacizumab therapy and may therefore be responsible for intratumoral vessel regression. In addition to programmed vessel regression, cessation of blood flow due to development of shunts or the thrombosis of tumor vessels can lead to tumor vessel regression via down-regulation of the transcription factor *kruppel*-*like factor2* (KLF2) and subsequent up-regulation of Ang-2 (Fig. 2b) [74, 145]. McDonald and colleagues have discussed the phenomenon of vasculogenic mimicry in detail and came to the conclusion that even in the prototype of cancer that shows vasculogenic mimicry, aggressive uveal melanomas, vascular channels are almost always lined by endothelial cells and not by cancer cells [98, 99], leading these authors generally doubt the significance of vasculogenic mimicry in tumor angiogenesis. Studies dealing with vasculogenic mimicry in human gliomas are sparse and mainly rely on PAS-staining to identify cancer cell-lined vascular channels. In one study, PAS-positive vascular channels devoid of CD34+ cells have been described in 2/45 human glioma specimens [155], and in 13 out of 101 samples in another study [89]. Recently, based on double-immunofluorescence staining, co-expression of GFAP (a marker for glioma cells) and VEGFR-2 (a marker for endothelial cells) has been reported in 7 out of 11 human GBM investigated and has been considered as evidence for vasculogenic mimicry [44], but this awaits confirmation. In summary, the functional and clinical relevance of vasculogenic mimicry appears questionable in light of the methodological problems encountered. Rather, we prefer to conclude from the data published that vasculogenic mimicry appears to be of little significance in glioblastoma angiogenesis, since most vessels appear to be lined by a proper endothelium on the luminal side.

*Bone marrow*-*derived vasculogenesis* describes the recruitment of circulating endothelial precursor cells to the tumor, their integration into the vessel wall and their terminal differentiation into an endothelial cell. From the onset of the original description of tumor vasculogenesis [2], this phenomenon has attracted a considerable amount of attention. This attention has stemmed from the fact that it has challenged the previous dogma that the postnatal vasculature (under both physiological and pathological conditions) can only proceed via angiogenesis but not vasculogenesis in order to adapt to altered physiological needs [121]. Mouse molecular studies that used chimeric mice with GFP-tagged bone marrow cells together with high-resolution confocal imaging and 3D-reconstruction revealed however that BM-derived endothelial cells in experimental gliomas represent only a small amount (less than 1 %) of all vascular endothelial cells [93]. However, it has been suggested that vasculogenesis is important for neovascularization after tumor irradiation [72]. Again, this issue has lead to a controversy since work from several laboratories suggests that bone marrow-derived cells do not incorporate into the vessel lumen to a significant extent but rather stay adjacent to the vessel, in a perivascular location [75]. These results suggest that bone marrow-derived cells support angiogenesis in a paracrine manner but presumably not by incorporation into the endothelial cell layer proper [76]. This imprecise use of the term “vasculogenesis” has facilitated the confusion in the literature about the process of tumor vasculogenesis [75]. Taken together, “real” tumor vasculogenesis appears to be a rare biological event that probably does not bear any clinical significance in human glioblastoma.

*Cancer stem-like cell derived vasculogenesis*: In 2010, two groups independently reported the transdifferentiation of GBM-derived stem-like cells into endothelial cells in vitro [120, 148]. The authors showed further that a proportion of vascular cells within human GBM contained genetic alterations that are typically encountered in glioblastoma cells (such as EGFR amplification) and are typically not seen in vascular endothelial cells. One group even reported that the majority of GBM vascular cells contained genetic alterations typically found in tumor cells [120]. The authors concluded that GBM-derived cancer stem-like cells can contribute to the vasculature by integrating into the vascular wall and transdifferentiate into endothelial cells, while retaining their genetic alterations. In contrast to this, recent work by Rodriguez [123] who examined GBM by detailed histological analysis demonstrated that in GBM (1) mutant vascular cells are extremely rare and (2) these cells are usually found in the perivascular space or vascular wall but were not lining the vessel lumen. Taken together, the existence of cancer stem-like cell-derived vasculogenesis in human glioblastoma is highly controversial and at best appears to be a very rare event.

The participation of *bone marrow derived cells in driving tumor angiogenesis* has achieved considerable attention recently (for reviews see [47, 66]). In particular, monocytes/macrophages can polarize into phenotypes that exert different functions in vivo. In contrast to M1 monocytes/macrophages, that are anti-tumorigenic, M2-polarized monocytes/macrophages are pro-angiogenic and thus drive tumor growth [135]. Current evidence suggests that monocytes/macrophages secrete a variety of pro-tumorigenic and pro-angiogenic growth factors (including VEGF). Experimental studies in glioma models suggest that blocking of monocyte/macrophage recruitment and/or function may block glioma growth (see below). As such, monocytes/macrophages represent an attractive target for anti-angiogenic tumor therapy although the relevance of bone marrow driven angiogenesis in human glioma still needs to be determined.

## Angiogenic signaling pathways in glioblastoma

Signaling pathways initiated from numerous growth factor receptors are known to play pivotal roles in developmental and tumor-mediated angiogenesis. In this review, we focus on three signaling pathways with a proven role in vascular biology and/or tumor angiogenesis that are currently being explored in clinical trials (Table 1) or represent major pharmaceutical targets that are under intense scrutiny. In addition to these angiogenic factors, further angiogenic signaling pathways are provided by axon guidance molecules that are known to play a role in both vascular and neuronal patterning (for recent review see [1, 37]. However, due to their prominent role in nervous system development and homeostasis it appears questionable whether these molecules will become targets for anti-angiogenic glioma therapy (see below and [115]).

**Table 1:** Current clinical trials targeting the VEGF/VEGFR or Ang/Tie2 signaling pathways in glioblastoma

Compound	Target	Combination	Phase	NCT number	Status	Company and/or trial center
Aflibercept	VEGF	Temozolomide Radiation	I	NCT00650923	Active	Sidney Kimmel Comprehensive Cancer Center
			II	NCT00369590	Completed	Sidney Kimmel Comprehensive Cancer Center
AZD2171 (Cediranib Maleate, Recentin)	VEGF	Bevacizumab	I	NCT00458731	Active	M.D. Anderson Cancer Center
Bevacizumab	VEGF	Temozolomide	II	NCT00590681	Active	Genentech
		Erlotinib	II	NCT00671970	Active	Genentech
		Irinotecan Voronistat	I	NCT00762255	Active	Merck
		Radiation Temozolomide	II	NCT01478321	Active	Northwestern University
		Temozolomide	II	NCT01013285	Active	UCLA
			II	NCT00271609	Active	National Cancer Institute
		Temsirolimus	II	NCT00800917	Completed	Roch/Pfizer
		Cetuximab Irinotecan	II	NCT00463073	Completed	
Bevacizumab	VEGF	Irinotecan	II	NCT00463203	Completed	
		TPI287	I/II	NCT01582152	Recruiting	Archer Biosciences, Inc.
		Voronistat	I/II	NCT01266031	Recruiting	Genentech
		Temozolomide	II	NCT01149850	Recruiting	Genentech
		Radiosurgery	I/II	NCT01086345	Recruiting	Case Comprehensive Cancer Center
		Lomustine	II	NCT01067469	Recruiting	M.D. Anderson Cancer Center
		Stereotactic Radiotherapy	I	NCT01392209	Recruiting	Genentech
		Lenalidomide	I	NCT01183663	Recruiting	M.D. Anderson Cancer Center
		Rindopepimut/GM-CSF	II	NCT01498328	Recruiting	Celldex Therapeutics
		Irinotecan	II	NCT00393094	Terminated	National Cancer Institute
AEE788	VEGFR, EGFR/ErbB2		I/II	NCT00116376	Completed	Novartis
BIBF 1120	VEGFR, PDGFR, FGFR		II	NCT01251484	Active	Boehringer Ingelheim Pharmaceuticals University of Copenhagen
Pazopanib	VEGFR-1/2/3, PDGFR, c-Kit	Lapatinib	II	NCT00350727	Completed	GlaxoSmithKline
AMG 386 (Trebanabnib)	Ang-1/2	Bevacizumab	I/II	NCT01290263	Suspended	Amgen
			I	NCT01538095	Recruiting	National Cancer Institute
		Bevacizumab	II	NCT01609790	Recruiting	National Cancer Institute (NCI)
PF- 04856884 (CVX-060)	Ang-2		II	NCT01225510	Withdrawn	Pfizer

*Ang* Angiopoietin, *VEGF* vascular endothelial growth factor, *VEGFR* vascular endothelial growth factor receptor

### VEGF signaling

VEGF is the principal angiogenesis factor in both embryonic development and tumor growth (for review see [38]). Deletion of either VEGF or VEGFR-1 or VEGFR-2 leads to embryonic lethality in the mouse due to severe defects in the developing vascular system [14, 42, 43, 133]. A role for VEGF in cerebral vascularization is illustrated by analysis of the conditional deletion of VEGF in the nervous system using a Nestin-cre/VEGF flox binary transgenic mouse to specifically delete VEGF in all Nestin-expressing cells. Mice carrying this deletion have a severe phenotype where the primitive perineural vascular plexus is able to develop, but vascular sprouts do not penetrate into the primitive avascular neuroectoderm. These mice have an avascular brain that causes microcephaly and massive periventricular apoptosis [116].

In GBM, VEGF is highly up-regulated, in particular in perinecrotic pseudopalisading cells [113, 134]. A major driving force of VEGF expression in GBM appears to be tumor hypoxia since VEGF and other hypoxia-inducible genes such as HIF-1 *alpha* and LDH are expressed in the same cell type [156]. Data derived from *The Cancer Genome Atlas (TCGA)* (http://cancergenome.nih.gov) suggest that in GBM the entire VEGF/VEGF receptor system is predominantly orchestrated by the up-regulation of VEGF; however, the VEGF receptors-1, -2 and -3 are also up-regulated in the tumor vasculature. The vast majority of clinical trials that target tumor angiogenesis use drugs that target the VEGF signaling pathway, either by blocking VEGF or by interfering with VEGFR-2 signaling (see Table 1 and for review [100]). The most advanced VEGF-specific drug currently on the market is bevacizumab, a monoclonal antibody that blocks VEGF function. Bevacizumab is routinely used in combination with chemotherapy (e.g., irinotecan) for the treatment of recurrent glioblastoma [19, 142, 147]. Unfortunately, several recent clinical reports suggest that anti-VEGF treatment may in fact promote a pro-migratory cellular tumor phenotype in patients with recurrent glioblastoma (for example [24, 102]). Importantly, these clinical observations are supported by several pre-clinical studies that in principle show that anti-VEGF treatment in mice or rats promotes a pro-invasive phenotype and may even increase tumor metastasis [35, 68, 107]. A possible explanation for this phenomenon, which represents a major challenge and clinical drawback, might be the induction of hypoxia and up-regulation of hypoxia-inducible genes via the HIF-1 *alpha* pathway [117]. Indeed, it has been suggested that hypoxia-mediated migration of tumor cells is responsible for the development of “pseudopalisading” glioma cells surrounding necroses, a histological hallmark of glioblastoma [10]. As such, these cells may represent glioma cells that are hypoxic and “try to escape” from a low oxygen microenvironment [110, 114]. The molecular mechanisms underlying this event are only partly understood. The protooncogene c-met, for example, has been shown to be up-regulated by hypoxia and to trigger invasion [9, 36, 108]. Recently, it has been suggested that c-met is activated in GBM upon bevacizumab treatment in a VEGFR-2 and phosphotyrosine phosphatase (PTP1B)-dependent manner. In this scenario, bevacizumab-induced depletion of VEGF reduces PTP1B activity and promotes c-met phosphorylation. This mechanism may therefore account at least in part for the observed therapy-induced switch to a pro-migratory phenotype after bevacizumab treatment [20, 92]. However, this putative mechanism requires the co-expression of c-met together with VEGFR-2 on glioma cells in vivo. Whereas VEGFR-2 can be readily detected in glioma cell lines in vitro, most studies suggest that in both murine and human glioma specimens in vivo expression of VEGFR-2 is mainly confined to vascular endothelial cells [51, 52, 77, 87, 112–114, 136], and potentially to a subset of cancer stem-like cells [49]. As such, it remains currently unclear whether the mechanism of bevacizumab-induced migration of glioma cells as proposed by Lu et al. is of relevance in human glioblastoma in vivo.

### Tie2/Angiopoietin signaling

**Fig. 3:**
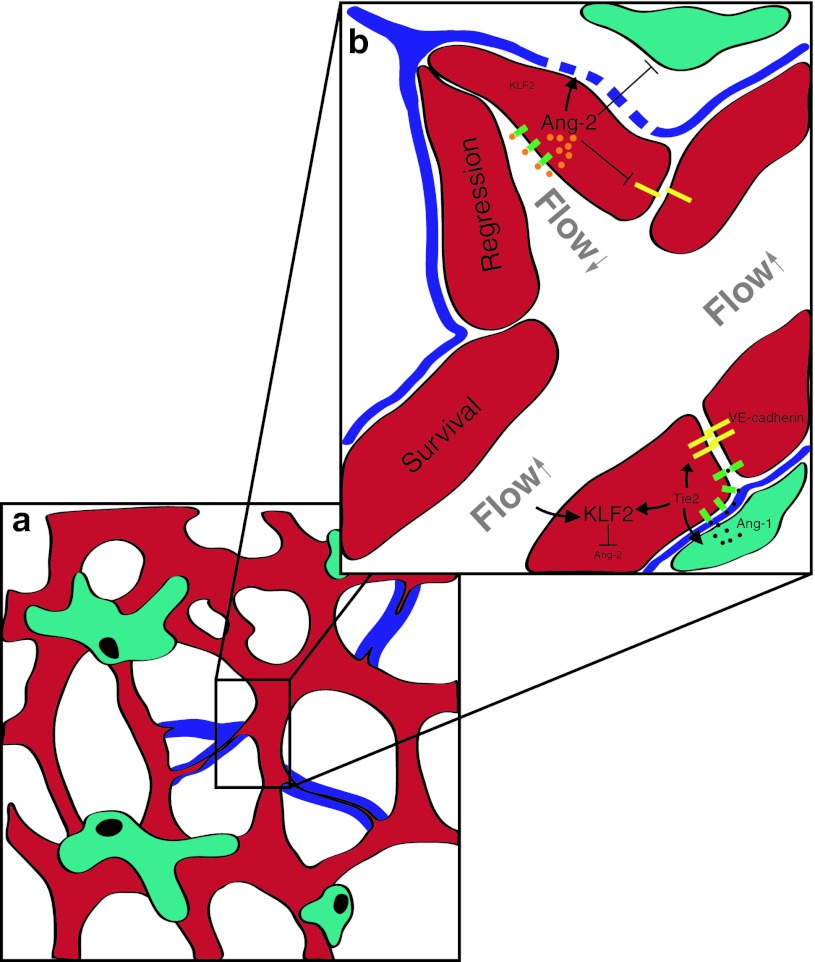
Involvement of Angiopoietins during vascular regression. **a** Vascular regression occurs under both, pathological and physiological settings and is characterized by local pericyte loss (turquois) and the disappearance of endothelial cells, leaving an empty basement membrane sleeve (“ghost vessels”/*blue*). **b** During vascular homeostasis shear stress is inducing Kruppel-like factor 2 (KLF2), a negative regulator of Angiopoietin-2 (Ang-2). Furthermore, Ang-1 (*black*) is secreted by mural cells into the extra cellular matrix (ECM/*blue*) and can then bind to endothelial expressed Tie2 (*green*) that will translocate to the cell–cell junctions upon Ang-1 binding forming homodimers in trans. Tie2 signaling further up-regulates KLF2 and stabilizes junctional molecules such as VE-cadherin (*yellow*). During vascular regression the occlusion of vessels leads to disturbed and reduced blood flow leading to KLF2 down-regulation. Ang-2 (*orange*) in turn becomes upregulated and triggers the dissociation of pericytes, the degradation of the basement membrane by inducing matrix metalloproteases (MMPs) and interferes with endothelial cell integrity

Pharmacological interference of the Tie2/Angiopoietin signaling pathway has received considerable attention recently (for review see [17, 58]). Several pre-clinical studies have shown that modulation of this pathway leads to alterations in vascular morphology and inhibition of tumor growth. Several types of angiopoietin inhibitors are now in phase I–III clinical trials (Table 1). The Tie2 receptor tyrosine kinase is expressed in endothelial cells and a subset of hematopoietic cells during development [34]. Similar to VEGF receptors, Tie2 is critical for normal vascular development [33]; however, in contrast to VEGF receptors that are down-regulated after embryonic angiogenesis has ceased, Tie2 appears to be constitutively expressed and phosphorylated in the adult vasculature [150]. This constitutive expression and activation suggests that signaling via Tie2 is important for the homeostasis of the mature vasculature (Fig. 3). Indeed, Tie2 signaling promotes stable vessels that are covered by pericytes. Tie2 is bound by three different ligands that engage the same binding site on the receptor. Angiopoietin-1 (Ang-1) and Angiopoietin-2 (Ang-2) are the first ligands to be discovered and are the best studied, whereas the function of Ang-4 is less understood. These ligands are thought to bind to Tie2 with roughly similar affinities and cause receptor activation [144]. In addition to Tie2, integrins serve as receptors for angiopoietins. A major observation has been that Ang-1 acts as a stimulating ligand for the Tie2 RTK that leads to receptor phosphorylation, whereas Ang-2 inhibits Tie2 phosphorylation, even in the presence of Ang-1 [64, 94]. Ang-1 can “seal” vessels in vivo and decrease vascular permeability [61]. Interestingly, the major regulator of the entire Tie2/Angiopoietin signaling pathway appears to be Ang-2. *TCGA* data for glioblastoma reveal that Ang-2 is up-regulated to a much higher extent than Ang-1, suggesting that Tie2 signaling is blocked in glioblastoma (http://cancergenome.nih.gov). The consequences of blocking Tie2 signaling in vivo, as evidenced by several pre-clinical studies, appears to be a shift towards an immature vasculature, whereas Ang-1 appears to “normalize” the vasculature (see below). In addition to an impact on angiogenesis, Ang-2 was shown to mediate a pro-inflammatory phenotype. In Ang-2 null mice the inflammatory response to stimuli such as TNF-α was greatly diminished when compared to controls [39]. Vice versa, in mice engineered to express an inducible form of Ang-2 in the vasculature, myeloid cells increased significantly over time in almost all organs, even without any pathological stimulus [128]. These findings suggest that Ang-2 on its own is able to orchestrate an inflammatory response by specifically recruiting myeloid cells—and leaving lymphocytes behind (reviewed in [69]). Moreover, since Ang-2 is highly expressed in glioblastoma vessels [141], Ang-2 up-regulation may also account—at least in part—for the attraction of myeloid cells of the monocyte/macrophage lineage to human gliomas. As such, inhibiting Ang-2 may have at least two different effects on tumor growth: (1) a shift of balance towards mature vessels (e.g., vascular normalization) and subsequent decrease of intratumoral hypoxia (2) inhibition of monocyte/macrophage recruitment to tumors [57, 59, 86, 97].

### Notch signaling pathway

The Notch signaling pathway plays an important role in organ development and recently has also received considerable attention in the vascular biology field recently (for review see [8, 78, 122]). Of the four Notch receptors, Notch1 and Notch4 are expressed on endothelial cells but Notch1 appears to be the most important for developmental angiogenesis. Similar to their receptors, Notch ligands are membrane-bound. Delta-like ligand4 (Dll4) appears to be the most important Notch ligand for stimulating angiogenesis, whereas another ligand, Jagged1, negatively regulates angiogenesis by competing with Dll4 [4, 78]. Notch in concert with VEGF signaling appears to be instrumental in the tip cell versus stalk cell fate decision that is essential for the initiation of sprouting angiogenesis (see Fig. 2). VEGF-A induces signaling in VEGFR-2 and -3, which are expressed in the tip cells leading to up-regulation of Dll4 and subsequent activation of Notch signaling in adjacent endothelial stalk cells. This signaling cascade places the Notch signaling pathway downstream of VEGFR-2 and R-3 signaling in endothelial cells. Once activated, Notch signaling can ultimately provide negative feedback on VEGFR signaling by up-regulating VEGFR-1 but inhibiting VEGFR-2 and R-3 expression in stalk cells, this signaling circuit provides a way for regulating tip versus stalk cell fate decisions [5]. The up-regulation of VEGFR-1, which acts as a decoy receptor, further contributes to the maintenance of the VEGF gradient. Blocking of Dll4–Notch interaction leads to hypervascular tumors that are nevertheless inhibited in their growth. This phenomenon has been entitled the “delta-paradox” and is probably based on the excessive production of non-functional vessels (e.g., tumor vessel abnormalization) [78]. Since Dll4 has been primarily detected in glioblastoma vasculature and not in glioma cells [65], targeting Dll4 by neutralizing antibodies is considered a potential anti-angiogenic tumor therapy. However, safety concerns have been arisen since it has been reported that in preclinical models, chronic Dll4 blockade abnormally activates endothelial cells and causes vascular neoplasms [152].

## The concept of vascular normalization

The concept of “vascular normalization” has been introduced by Jain [60]. This concept states that anti-angiogenic (e.g., Bevacizumab) treatment merely affects the immature vasculature and leaves the mature vessels unaltered. Indeed, it has been shown that VEGF withdrawal leads to selective ablation of immature blood vessels [6]. As such, a “normalized” vasculature results as a consequence of anti-VEGF treatment, leading to increased perfusion of the tumor and subsequent increase of oxygenation. Vascular normalization is thought to interrupt the vicious circle that is driven by hypoxia and that leads to up-regulation of VEGF, resulting in the growth of immature—partly unperfused—vessels and a subsequent increase in tumor hypoxia [23]. Some researchers believe that vessel normalization followed by cytotoxic chemotherapy and/or radiotherapy should be the ultimate goal of any anti-angiogenic therapy [62]. Indeed, in an experimental glioma model, intratumoral vascular normalization restored blood–brain barrier properties by suppressing edema formation [118] and a clinical study showed increased blood perfusion in GBM after anti-angiogenic therapy [138]. In order to achieve vascular normalization, several potential targets beside anti-VEGF therapy have been identified that include (1) Tie2/Angiopoietin signaling (see above), (2) prolylhydroxylases, which are upstream of the HIF-signaling pathway [23], (3) blockade of the VEGF receptor-1 ligand placenta growth factor (PlGF) [40], (4) blocking of TGF-ß signaling [31] and (5) increase of vascular wnt/ß-catenin signaling [118].

## The role of myeloid cells in tumor angiogenesis

**Fig. 4:**
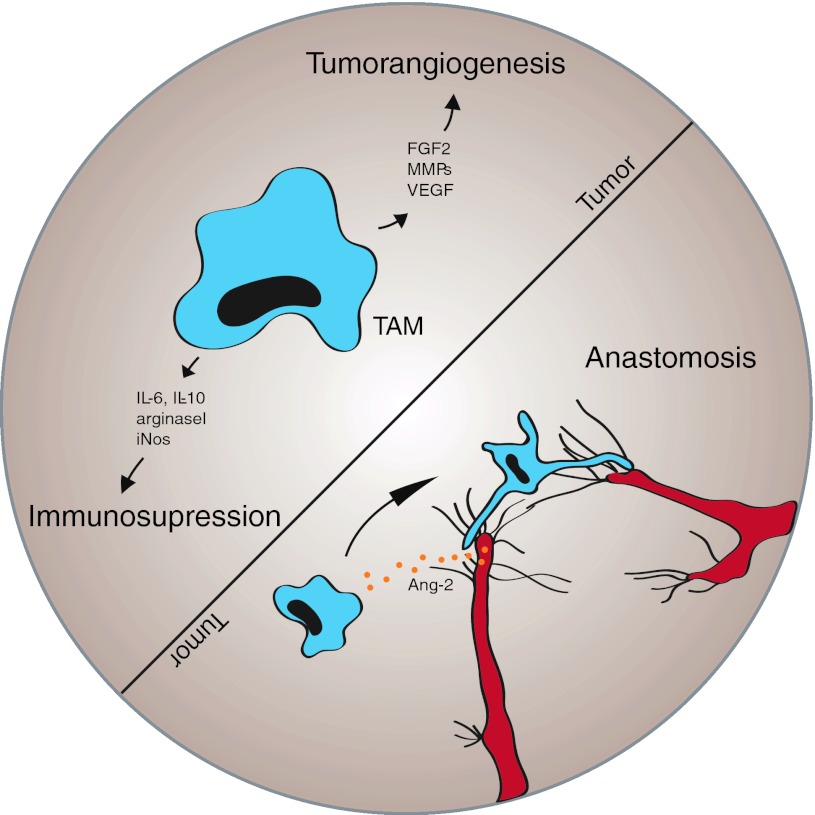
The role of tumor-associated macrophages (TAMs) during tumor angiogenesis. TAMs (*blue*) can promote tumor growth in at least two different ways: 1. TAMs, actively suppress the hosts immune response to tumors by secretion of immunosuppressive mediators, such as IL-6 and IL-10. 2. TAMs are a significant source of angiogenic factors (e.g., FGF2, MMPs, VEGF) that promote tumor neovessel formation and thereby contribute to malignant progression. Macrophages are thought to participate in and support the process of anastomosis, the fusion of two vascular sprouts to establish a direct connection. During this setting, macrophages (*blue*) can be found intimately associated with endothelial tip cells (*red*). It is hypothesized that their recruitment to sites of vessel fusion is driven by tip cell secreted Ang-2 (*orange*) that can attract macrophages in a Tie2-dependent or Tie-2 independent pathway, with the latter being mediated by beta2-integrins

Current studies suggest that infiltrating myeloid cells contribute significantly to tumor angiogenesis, presumably by secreting pro-angiogenic factors including VEGF, stromal-derived factor-1 (SDF-1) and others (Fig. 4, for review see [47, 101]). Grunewald et al. [48] have shown that myeloid cells are (1) recruited to tumors, (2) “educated” by the tumor microenvironment and (3) support tumor growth by secretion of SDF-1. In another study, chimeric mice were generated by transplanting bone marrow from VEGFR-1 signaling deficient mice into lethally irradiated wild-type mice. When Gl261 glioma cells were transplanted intracranially into these mice, tumor growth was significantly inhibited when compared to mice harboring a normal bone marrow. Subsequent studies showed that VEGFR-1 signaling deficient bone marrow chimeras displayed a defect in monocyte migration leading to significant lower amount of myeloid cells in the tumor tissue [67]. In a preclinical study, myeloid cell infiltration has been shown to be associated with tumor progression after anti-angiogenic therapy [109]. In a clinical study, a decrease in the number of VEGFR-1 + monocytes in the blood correlated with improved response after bevacizumab treatment [25]. Most of the myeloid cells that support tumor growth through the stimulation of angiogenesis appear to be monocytes/macrophages with M2 polarity (TAMs, tumor-associated macrophages) [135]. In contrast, bone marrow-derived non-hematopoietic mesenchymal stem cells may in fact suppress glioma growth through the inhibition of angiogenesis [54]. Interestingly, one distinct subtype of monocytes with particular importance for tumor angiogenesis and immunosuppression seems to be Tie2-expressing monocytes (TEMs) [28]. TEMs represent a subset of circulating blood monocytes that are recruited from the circulation into tumors and locally support tumor growth in a paracrine manner [88]. Although the number of TEMs within a tumor appears relatively small (e.g., <10 % of infiltrating monocytes express Tie2) [21], loss of function studies using suicide gene approaches have shown that the specific depletion of TEMs is able to block tumor angiogenesis and tumor growth [29]. The number of infiltrating macrophages has been shown to be of prognostic relevance in breast cancer [85], rectal cancer [132], classic Hodgkin’s lymphoma [139], adenocarcinoma of the lung [157] and hepatocellular carcinoma [27, 96]. Therefore, both TEMs and TAMs clearly have evolved as novel targets for anti-angiogenic cancer therapy [26, 66].

## Future prospects for anti-angiogenic therapy

The first specific anti-angiogenic drug that received FDA approval in 2004 was bevacizumab, a neutralizing monoclonal antibody to VEGF. Bevacizumab is widely used in different types of cancer and significantly inhibits tumor growth in many cancer types (see Table 1 for current anti-angiogenesis trials in glioblastoma). However, drugs that interfere with the function of the vascular system may also cause—sometimes severe—undesired effects [3, 84]. VEGFR-2 is not only expressed on endothelial cells but also on hemangioblasts and angioblasts [121] and in the nervous system (see below). Documented undesired effects of bevacizumab therapy include hypertension, induction of bleeding disorders and deep venous thrombosis [100, 103]. Chronic inhibition of the Notch pathway by Dll4 blockade using inhibitory antibodies may even lead to the induction of vascular neoplasms [152]. In addition, many vascular growth factors have been shown to be involved in the development and homeostasis of the nervous system by directly interacting with neurons (for review see [37, 115, 124, 125]). VEGF, for example, acts as a survival factor for neurons in vitro and in vivo and promotes neurogenesis, most likely through interaction with VEGFR-2 expressed on neurons [13, 22, 63, 127, 149, 153]. Partial deletion of VEGF in mice leads to loss of motoneurons and subsequently to a phenotype that highly resembles amyotrophic lateral sclerosis (ALS) in humans [106]. Indeed, polymorphisms in the *vegfa* gene are associated with a higher risk to develop ALS in humans [83] and delivery of recombinant VEGF has been used successfully to protect motoneurons in a rat model of ALS [140]. Currently, intracerebroventricular delivered VEGF is in clinical phase I/II trial for patients with ALS. Further, in line with a direct effect of VEGF on the nervous system, anti-VEGF receptor treatment has been reported to lead a painful sensory neuropathy [146].

In addition to the above-mentioned adverse effects of anti-VEGF treatment, pre-clinical and clinical reports suggest that cancer cells may develop resistance to anti-angiogenic therapy by different mechanisms that include (1) switch to a pro-migratory phenotype, (2) up-regulation of other pro-angiogenic molecules [151] and (3) the increased recruitment of myeloid cells that support tumor growth (reviewed in [7, 66, 129]). As such, novel therapies are currently under development in both academia and pharmaceutical companies that include (1) novel VEGF inhibitors such as the VEGF trap [46, 55], (2) drugs that target the Tie2/Angiopoietin signaling pathway (reviewed in [17, 58]), (3) double inhibition of VEGF and angiopoietins [12, 18, 73], (4) double inhibition of VEGF and c-met [130, 154], (5) inhibition of PlGF [30, 40] and (6) drugs that block myeloid cell recruitment and/or their polarization [91], among others. However, it has been suggested that intratumoral vessel are heterogenous and that, for example, VEGF-dependent and -independent vessels may co-exist within a given tumor [137]. These observations speak in favor of a multi-modal therapy that may combine different types of anti-angiogenic therapy or anti-angiogenic therapy together with cytotoxic therapy. The integrin inhibitor cilengitide, for example, targets both tumor cells and the vasculature [105], and is now in phase III clinical trial for glioblastoma patients [79, 126]. In our personal view, targeting the Tie2 signaling pathway by blocking Ang-2 or stimulating Ang-1 (or both simultaneously) may be particularly promising since three mechanisms of tumor angiogenesis may be targeted similarly: (1) sprouting angiogenesis and (2) myeloid cell infiltration may be blocked, whereas (3) vascular normalization will be enhanced [17, 58]. Further, reported side effects of Ang-2 blocking appear to be sparse compared to other anti-angiogenic therapies employed [53, 82]. However, this has to be proven further in the clinical trials that are currently under way. It may also be necessary to stratify patients so that those that have a shift in the Ang-1/Ang-2 balance or other angiogenic factors are identified and the appropriate therapy initiated. For example, the monitoring of expression patterns of angiogenesis factors and their receptor in the tumor biopsy or the evaluation of biomarkers in the serum or cerebrospinal fluid of patients may provide useful indicators of a tumor’s susceptibility to a particular targeted therapy [100]. Promising biomarkers that may indicate therapy responsiveness in anti-angiogenic therapy include serum Ang-2 levels and the number of circulating VEGFR-1 + monocytes [25, 45, 90]. It will be exciting to watch whether any of the newly developed therapies will be able to significantly affect survival of brain cancer patients.
